# Maritime Pine Rootstock Genotype Modulates Gene Expression Associated with Stress Tolerance in Grafted Stems

**DOI:** 10.3390/plants13121644

**Published:** 2024-06-14

**Authors:** Lorenzo Federico Manjarrez, María Ángeles Guevara, Nuria de María, María Dolores Vélez, Irene Cobo-Simón, Miriam López-Hinojosa, José Antonio Cabezas, José Antonio Mancha, Alberto Pizarro, María Carmen Díaz-Sala, María Teresa Cervera

**Affiliations:** 1 Departamento de Ecología y Genética Forestal, Instituto de Ciencias Forestal (ICIFOR), Instituto Nacional de Investigación y Tecnología Agraria y Alimentaria—Consejo Superior de Investigaciones Científicas (INIA–CSIC), 28040 Madrid, Spain; lorenzo.federico@inia.csic.es (L.F.M.); ndemaria@inia.csic.es (N.d.M.); velez.mdolores@inia.csic.es (M.D.V.); irene.cobo@inia.csic.es (I.C.-S.); mirialca_92@hotmail.com (M.L.-H.); cabezas.joseantonio@inia.csic.es (J.A.C.); mancha.jose@inia.csic.es (J.A.M.); 2 Departamento de Ciencias de la Vida, Universidad de Alcalá (UAH), 28805 Alcalá de Henares, Spain; alberto.pizarro@uah.es (A.P.); carmen.diazsala@uah.es (M.C.D.-S.)

**Keywords:** *Pinus pinaster*, grafting, transcriptomic analysis, drought tolerance, genotype selection, stem

## Abstract

Climate change-induced hazards, such as drought, threaten forest resilience, particularly in vulnerable regions such as the Mediterranean Basin. Maritime pine (*Pinus pinaster* Aiton), a model species in Western Europe, plays a crucial role in the Mediterranean forest due to its genetic diversity and ecological plasticity. This study characterizes transcriptional profiles of scion and rootstock stems of four *P. pinaster* graft combinations grown under well-watered conditions. Our grafting scheme combined drought-sensitive and drought-tolerant genotypes for scions (GAL1056: drought-sensitive scion; and Oria6: drought-tolerant scion) and rootstocks (R1S: drought-sensitive rootstock; and R18T: drought-tolerant rootstock). Transcriptomic analysis revealed expression patterns shaped by genotype provenance and graft combination. The accumulation of differentially expressed genes (DEGs) encoding proteins, involved in defense mechanisms and pathogen recognition, was higher in drought-sensitive scion stems and also increased when grafted onto drought-sensitive rootstocks. DEGs involved in drought tolerance mechanisms were identified in drought-tolerant genotypes as well as in drought-sensitive scions grafted onto drought-tolerant rootstocks, suggesting their establishment prior to drought. These mechanisms were associated with ABA metabolism and signaling. They were also involved in the activation of the ROS-scavenging pathways, which included the regulation of flavonoid and terpenoid metabolisms. Our results reveal DEGs potentially associated with the conifer response to drought and point out differences in drought tolerance strategies. These findings suggest genetic trade-offs between pine growth and defense, which could be relevant in selecting more drought-tolerant *Pinus pinaster* trees.

## 1. Introduction

Hazards resulting from ongoing climate change threaten the balance of ecosystems. Aggravated ecological disturbances, such as recurrent and severe droughts, high temperatures, and associated forest fires and pest outbreaks, continuously challenge their resilience [1]. An increasing number of studies have shown that the Mediterranean Basin is one of the most vulnerable areas to suffer from these types of disturbances, especially its forest ecosystems. Forests are hotspots of biodiversity and providers of many ecosystem services that preserve human well-being. Climate change-induced disturbances severely increase the decline of Mediterranean forests and consequent losses in forest productivity and biodiversity [2,3,4,5]. Therefore, forest management programs in this region must improve forest resilience to address deforestation and forest degradation while aiming to sustainably meet the growing demand for timber and wood products [6,7].

Maritime pine (*Pinus pinaster* Aiton) is a native conifer representative of western Mediterranean forests that has been used, for decades, in numerous reforestation programs [8], expanding its distribution. Despite its limited natural distribution, maritime pine shows a remarkable genetic and phenotypic diversity as well as ecological plasticity that has made it a model species in south-western Europe [9,10]. This ecological plasticity has enabled maritime pine to populate diverse forest systems throughout its range. Numerous studies have identified a remarkable variation in high-value traits such as growth rate [11], biomass allocation [12], stem shape [13], wood quality [14], metabolite content [15], drought response [16,17], and resistance to pests and diseases [10,18,19].

Clonal propagation of forest species is mainly achieved via somatic embryogenesis, the rooting of cuttings, and organogenesis [20]. However, for some forest species, it is limited to the early stages of their development. This is the case for *P. pinaster* whose regenerative capacity decreases during the first years of development, reducing the adventitious rooting rate of cuttings [21]. A suitable alternative method for clonally propagating recalcitrant species is grafting, which has been used for centuries in the production of many woody fruit trees and horticultural crops. Grafts are mainly designed to combine scions and rootstocks of genotypes with desirable phenotypes, requiring compatibility between them. Grafting is commonly used for the vegetative propagation of elite fruit tree and vegetable genotypes, avoiding the juvenility stage by grafting young scions onto rootstocks from mature trees. Thus, this strategy increases productivity and the combination of elite scions with selected compatible rootstocks improves scion fitness, including graft response to biotic or abiotic stresses [22]. In conifers, such as maritime pine, grafting is mainly used to preserve genotypes with high-value traits and to produce high-quality seeds from superior individuals in clonal seed orchards, using cuttings as scions and unselected rootstocks grown from seeds, but is rarely used for mass propagation [23,24]. However, the selection of elite trees resistant to new pest outbreaks and diseases, as well as abiotic stresses such as recurrent drought periods will make it necessary to improve the use of grafting for propagation as well as the development of rootstock breeding programs.

Nowadays, grafting has become a useful technique to study how rootstocks affect scion performance, as well as the biological mechanisms underlying long-distance communication and transport of graft-mobile molecules between organs. Grafted plants maintain the genetic identity of each part, but mobile molecules, such as proteins, small peptides, mRNA, or miRNA, are transported across the junction from one to the other [25,26]. Most studies have been conducted in angiosperms, mainly in model plants, such as Arabidopsis, or crop species, in which the importance of genotype selection for both scion and rootstock has been highlighted [27,28,29,30]. In Arabidopsis, grafting has led to the identification of numerous graft-mobile molecules that regulate a wide range of biological processes. Among these, there are proteins, such as the FLOWERING LOCUS T (FT) which is transported from leaves to shoots to induce flowering [31], and small peptides such as CLE25, which is transported from root to shoot in response to drought stress, triggering ABA-induced stomatal closure [32]. In addition, studies involving interspecific hetero-grafts, such as Arabidopsis grafted with tobacco (*Nicotiana benthamiana*), have revealed several graft-mobile mRNAs, including transcription factors (ICE2, BEL10, BSH) and cell wall modifiers (arabinogalactan proteins 15 and 23) [33]. In potato (*Solanum tuberosum*), most grafting studies have focused on the regulation of tuberization, describing graft-mobile proteins (e.g., FLOWERING LOCUS T [34] and CONSTANS) [35], mRNA (e.g., BELLRINGER1-like BEL5 [36], BELL11, and BEL29 [37]), or miRNA (e.g., miR156 [38] and miR172 [39]).

Nevertheless, the number of studies on forest trees, particularly conifers, is scarce. Moreover, due to the divergence between gymnosperms and angiosperms 140–270 Mya [40,41] and their morphological and functional differences, particularly in vascular systems (tracheids vs. vessels) [42], functional information from grafts in flowering plants cannot be applied to conifers. Recent studies on maritime pine grafts have shown differences in their physiological and metabolic responses to drought [43]. In addition, rootstock genotype has been shown to significantly modify the transcriptomic profile of scion needles in maritime pine grafts, where rootstock genotypes with contrasting drought response could have regulated the accumulation of transcripts associated with drought response prior to water deprivation [44]. Therefore, a similar modification in the transcriptomic profiles of both scion and rootstock stems may occur, influenced by factors such as genotype, stem type, or genotype combination. These factors could be crucial for drought response in *P. pinaster* stems, an organ not typically considered when studying drought responses, but which potentially plays a significant role.

For this study, we analyze the transcriptional profiles of scion and rootstock stems, the organ that ensures the efficient transport of water, inorganic and organic compounds in the open vascular system of trees, and the communication of any functional graft to identify variations that could be associated with the effect of each genotype in this chimeric organ.

## 2. Results

### 2.1. Sequencing and Annotation of Stem Transcriptome

A total of 24 stem samples were used for RNA-seq, two stem samples (scion and rootstock) for each one of the three biological replicates (grafts) for the four types of constructs: Gal 1056/R1S (S_S_/S_R_), Gal 1056/R18T (S_S_/T_R_), Oria 6/R1S (T_S_/S_R_), and Oria 6/R18T (T_S_/T_R_). Sequencing of the 24 libraries yielded 1087.486 million 151 bp paired-end reads (Table S1) with a mean Q30 of 95%. The number of paired-end reads after trimming and filtering was 513.565 million. Approximately, 93.27% (91.81–94.13%) of them aligned with the *P. pinaster* reference transcriptome (Table S2).

BLASTx mapping of the *P. pinaster* reference transcriptome with the non-redundant NCBI, Swiss-Prot, and InterPro databases resulted in 70,086 aligned transcripts out of 206,575 (33.92%). Gene Ontology (GO) terms were assigned to a total of 69,532 blasted sequences, of which 17,039 were classified into 253 KEEG metabolic pathways. For comparisons, we refer to the stem (scion and/or rootstock) analyzed in italics. A total of 14,253 transcripts from the *P. pinaster* reference transcriptome were differentially expressed in scion and rootstock stems. The total number of DEGs mapped by BLAST was 9497 (66.63%), and 8551 (59.99%) had GO terms assigned. The percentage of annotated sequences ranged from 70.69% (*S_S_*/S_R_ vs. *S_S_*/T_R_) to 57.61% (*S_S_*/T_R_ vs. *T_S_*/T_R_) (Figure S1), and 1874 sequences were linked to at least one of the 137 KEGG metabolic pathways identified.

### 2.2. Variations among Graft Types: Differential Expression Analysis

**Figure 1:**
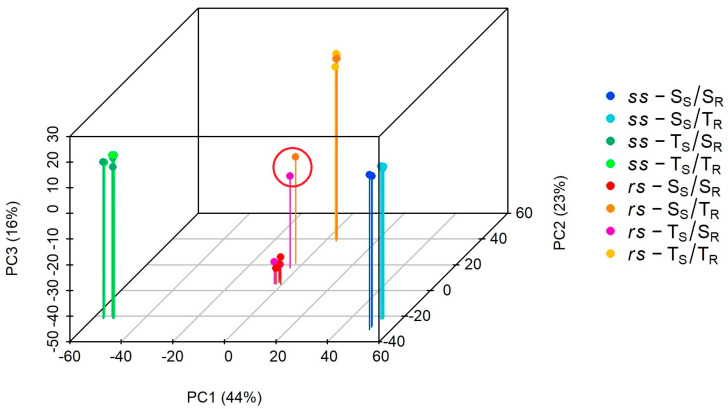
Principal component analysis performed on scion (ss) and rootstock (rs) stems of *Pinus pinaster* grafts. The analysis considered the top 1000 genes. Two samples from rootstock stems, marked with a red circle, were removed from further analysis. The removed samples were from the T_S_/S_R_ and S_S_/T_R_ constructs.

Principal component analysis (PCA) revealed that the main differences among samples were associated with their genotype (Figure 1).

**Figure 2:**
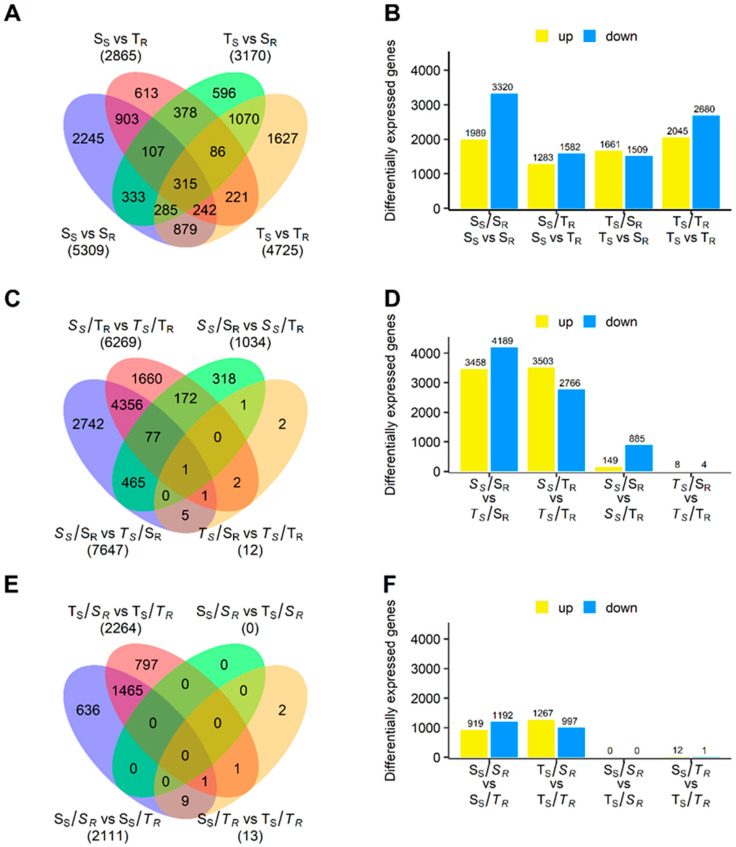
Differential expression analysis in *Pinus pinaster* graft stems. (**A**) The distribution of total DEGs identified in comparisons between scion and rootstock stems of each graft construction, and (**B**) their classification according to their regulation. (**C**) The distribution of total DEGs identified in comparisons of scion stems with contrasting drought response (blue and red), drought-sensitive scion stems (green), and drought-tolerant scion stems (yellow), and (**D**) their classification according to their regulation. (**E**) The distribution of DEGs identified in the comparison of rootstock stems with contrasting drought response (blue and red), drought-sensitive rootstock stems (yellow), and drought-tolerant rootstock stems (green), and (**F**) their classification according to their regulation.

**Scheme 1:**
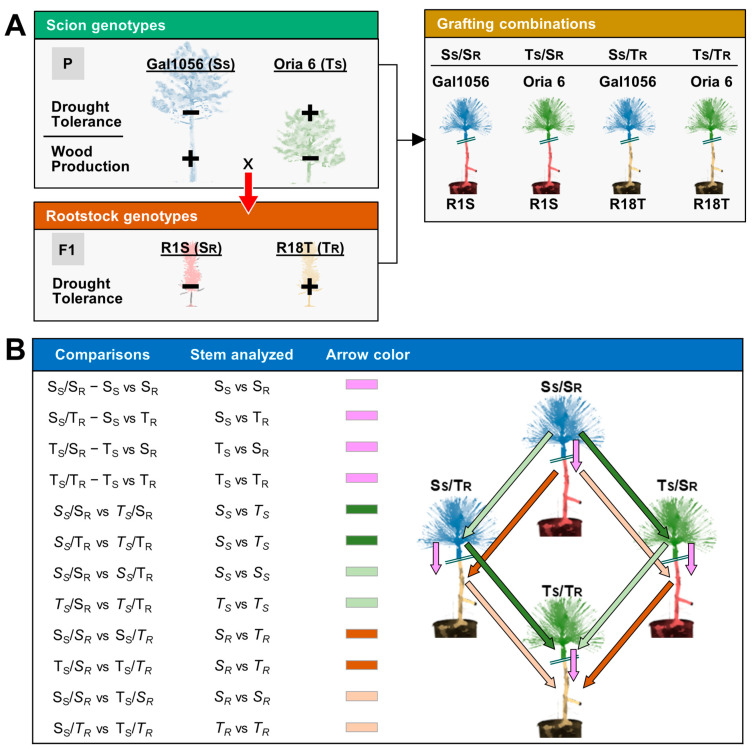
Graft design and stem comparisons. (**A**) Scion and rootstock stem samples were collected from *Pinus pinaster* grafts combining four genotypes with contrasting drought tolerance. Gal1056 and Oria 6 were the donors of drought-sensitive (S_S_) and drought-tolerant (T_S_) scions, respectively. R1S and R18T, two full-sibs from the controlled cross Gal1056 x Oria 6, were used as drought-sensitive (S_R_) and drought-tolerant rootstocks (T_R_). The four designed grafts were Gal 1056/R1S (S_S_/S_R_), Gal 1056/R18T (S_S_/T_R_), Oria 6/R1S (T_S_/S_R_), and Oria 6/R18T (T_S_/T_R_). At least three biological replicates were used for each type of graft. (**B**) Differential analysis between scion stems (green) and rootstocks (brown) or between scions and rootstocks (pink). Arrows indicate the direction of the comparison, pointing to the samples used as tests in each comparison: A → B (A vs. B). Stems (from scion and/or rootstock) tested are in italics. Pink arrows indicate comparisons between scion and rootstock stems of each *P. pinaster* graft that combined genotypes with similar or contrasting responses to drought (S vs. R stems). Green and brown arrows indicate comparisons between scions and rootstock stems, respectively. The dark green and brown arrows indicate the comparisons conducted to identify differentially expressed genes (DEG) between drought-sensitive and drought-tolerant genotypes in scions and rootstock stems, respectively (Contrasting tolerance). Their light-colored alternatives indicate the comparisons conducted to identify DEGs associated with the effect of rootstock or scion genotypes on scion and rootstock stems, respectively (Genotype effect).

A total of twelve comparisons between scion and/or rootstock stems were conducted, as illustrated in Scheme 1. Comparisons between different scions grafted onto drought-sensitive (*S_S_*/S_R_ vs. *T_S_*/S_R_) and drought-tolerant (*S_S_*/T_R_ vs. *T_S_*/T_R_) rootstocks showed the highest number of DEGs, 7647 and 6269, respectively (Figure 2C). In the *S_S_*/S_R_ vs. *T_S_*/S_R_ comparison, a higher number of down-regulated DEGs was observed as a result of the higher transcript accumulation in the drought-sensitive scions from *S_S_*/S_R_ grafts (Figure 2D). In addition, *S_S_*/*S_R_* grafts showed the highest number of DEGs (5309 DEGs) when comparing the transcriptomes of scion and rootstock stems of each construct (Figure 2A). In scion stems, the comparison between drought-sensitive scions grafted onto both types of rootstocks (*S_S_*/S_R_ vs. *S_S_*/T_R_) showed 1034 DEGs, of which 885 were more accumulated in scions grafted onto drought-sensitive rootstocks. However, only 12 DEGs were identified in drought-tolerant scions grafted onto both types of rootstocks (*T_S_*/S_R_ vs. *T_S_*/T_R_) (Figure 2C,D). The comparisons that included the lowest number of DEGs were those between drought-sensitive (S_S_/*S_R_* vs. T_S_/*S_R_*, with no DEGs identified) or drought-tolerant rootstock (S_S_/*T_R_* vs. T_S_/*T_R_*, with 13 DEGs) stems grafted with both types of scions (Figure 2E,F).

### 2.3. Differences between Scion and Rootstock Stems from the Same Graft

Transcript profiles of both stem samples obtained from each graft were compared. These comparisons included grafts combining scions and rootstocks with a similar response to drought, either sensitive (S_S_/S_R_—*S_S_* vs. *S_R_*) or tolerant (T_S_/T_R_—*T_S_* vs. *T_R_*), as well as grafts combining scions and rootstocks with a contrasting response to drought (S_S_/T_R_—*S_S_* vs. *T_R_* and T_S_/S_R_—*T_S_* vs. *S_R_*).

**Figure 3:**
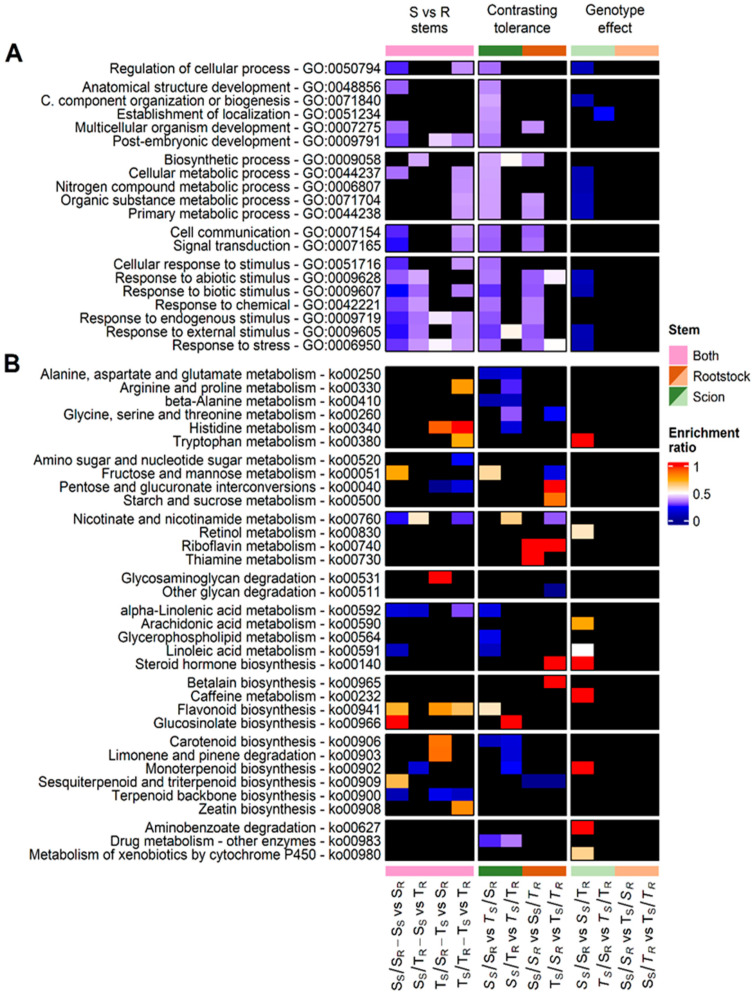
(**A**) Enrichment analysis of GO terms. (**B**) Enrichment analysis of KEGG metabolic pathways. Significantly enriched Go and KEGGs categories had FDR > 0.05 and *p*-value < 0.05, respectively. Absence of significant data is represented in black. Enrichment ratio closer to one (in red) indicates DEG overrepresentation in rootstock stems, drought-tolerant genotypes, or in stems grafted with the drought-tolerant genotype in S vs. R stems, Contrasting tolerance, or Genotype effect columns, respectively. In contrast, enrichment ratio closer to zero (in blue) indicates DEG overrepresentation in scion stems, drought-sensitive genotypes, or in stems grafted with the drought-sensitive genotype in S vs. R stems, Contrasting tolerance or Genotype effect columns, respectively.

The highest number of DEGs was identified in scion and rootstock stems from grafts combining genotypes with a similar drought response, S_S_/S_R_ and T_S_/T_R_ (Figure 2A), which was mainly associated with a lower accumulation of transcripts in their rootstock stems. There were 3320 DEGs (62.53%) in S_S_/*S_R_* and 2680 DEGs (62.69%) in T_S_/*T_R_* (Figure 2B). Functional enrichment analysis revealed that 18 out of 20 GO terms were significantly overrepresented (FDR < 0.05) among scion vs. rootstock stem comparisons, with the highest number of DEGs in scion stems of the *S_S_*/S_R_, *T_S_*/T_R_, and *S_S_*/T_R_ grafts (Figure 3A—S vs. R stems).

Focusing on enriched categories in scion stems (enrichment ratio is closer to zero: blue), the drought-sensitive scion stems (*S_S_*) of *S_S_*/S_R_ grafts showed enriched terms associated with stress and stimulus responses, communication, and development. Specific GO terms overrepresented in drought-tolerant scion stems (*T_S_*) of *T_S_*/T_R_ grafts were mainly associated with primary metabolic processes (Figure 3A—S vs. R stems). MapMan (version 3.7.0) analysis showed a significant accumulation of transcripts belonging to the AP2/EREBP (BIN 27.3.3) and MADS-box (BIN 27.3.24) transcription factor (TF) families in drought-sensitive scion stems (*S_S_*) from *S_S_*/S_R_ grafts (Table S3). In drought-tolerant scion stems (*T_S_*) from *T_S_*/T_R_ grafts, transcripts associated with terpenoids metabolism (BIN 16.1.5), gibberellin synthesis–degradation (copalyl diphosphate synthase, BIN 1.6.1.1), ADH enzymes (BIN 26.11), biotic stress (BIN 20.1), and PR proteins (BIN 20.1.7) were significantly enriched (Table S3). Analysis of KEGG metabolic pathways showed enriched categories (*p*-value < 0.05) in scion stems, particularly, in scion stems of *S_S_*/S_R_ and *T_S_*/T_R_ grafts. Common metabolic pathways in scion stems were involved in linoleic acid metabolism (ko00591 and ko00592), terpenoid backbone biosynthesis (ko00900), and nicotinate and nicotinamide metabolism (ko00760; Figure 3B—S vs. R stems).

In the case of rootstock stems (enrichment ratio is closer to one: red), no significant GO category stood out in these comparisons (Figure 3A—S vs. R stems). However, MapMan and KEGG analyses revealed enriched clusters in rootstock stems. KEGG analysis revealed a higher accumulation of transcripts associated with flavonoid biosynthesis in the rootstock stems of S_S_/*S_R_*, T_S_/*S_R,_* and T_S_/*T_R_* grafts (Figure 3B—S vs. R stems). Furthermore, enriched clusters in the drought-sensitive rootstock stems (*S_R_*) of the S_S_/*S_R_* grafts were associated with photosystems (BIN 1) and light-harvesting complex II (LHCII, BIN 1.1), cytochrome P450 (BIN 26.1), lignin biosynthesis (BIN 16.2.1), and biosynthesis of flavonoids (ko00941), such as dihydroflavonols (BIN 18.8.3) (Table S3). In the drought-tolerant rootstock stems (*T_R_*) of T_S_/*T_R_* grafts, the clusters that included the highest number of DEGs were related to the metabolism of amino acids, such as proline, histidine, and tryptophan (ko00330, ko00340, and ko00380), zeatin biosynthesis (ko00908) (Figure 3B—S vs. R stems), and the metabolism of secondary metabolites such as phenylpropanoids (lignin biosynthesis, COMT: BIN 16.2.1.9) and flavonoids (BIN 16.8; chalcones: BIN 18.8.2; and dihydroflavonols/flavonoid-3-monooxygenase: BIN 16.8.3.3) (Figure 3B—S vs. R stems and Table S3).

### 2.4. Identification of DEGs Associated with Differences between Drought-Sensitive and Drought-Tolerant Scion (S_S_ vs. T_S_) and Rootstock (S_R_ vs. T_R_) Stems

These comparisons provided valuable information on transcriptional differences between different scions (*S_S_* vs. *T_S_*) grafted onto the same rootstocks (*S_S_*/S_R_ vs. *T_S_*/S_R_ and *S_S_*/T_R_ vs. *T_S_*/T_R_), as well as different rootstocks (*S_R_* vs. *T_R_*) grafted with the same scion type (S_S_/*S_R_* vs. S_S_/*T_R_* and T_S_/*S_R_* vs. T_S_/*T_R_*). A higher number of DEGs was identified in comparisons between scion stems than between rootstock stems (Figure 2C,E). In particular, the highest level of gene expression was identified in drought-sensitive scion stems (*S_S_*) in the *S_S_*/S_R_ vs. *T_S_*/S_R_ comparison (Figure 2D).

#### 2.4.1. Comparisons among Scion Stems with Contrasting Drought Tolerance (S_S_ vs. T_S_): S_S_/S_R_ vs. T_S_/S_R_ and S_S_/T_R_ vs. T_S_/T_R_

Overrepresented GO terms were only found in drought-sensitive scion stems (*S_S_*) while grafted onto drought-sensitive rootstocks (S_R_) in the *S_S_*/S_R_ vs. *T_S_*/S_R_ comparison. Those GO terms included a significant number of transcripts related to stress response, development, metabolism, stimulus response, communication, and signaling (Figure 3A—Contrasting tolerance). In addition, MapMan analysis showed that DEGs involved in transcriptional regulation (BIN 27.3), AP2/EREBP TFs (BIN 27.3.3), and signaling through legume lectin (BIN 20.2.19) and LRR-RK XI (BIN 30.2.11) receptor kinases were significantly overrepresented in sensitive scion stems of *S_S_*/S_R_ (Table S4).

Metabolic pathways analysis revealed several metabolic pathways significantly enriched in drought-sensitive scion stems (S_S_), regardless of the rootstock genotype. These pathways included beta-alanine (ko00410), alanine, aspartate, and glutamate (ko00250) metabolism, as well as carotenoid biosynthesis (ko00906). In addition, other metabolic pathways were identified as significantly enriched in drought-sensitive scion stems (S_S_) when grafted onto drought-tolerant rootstocks. These included monoterpenoid biosynthesis (ko00902) and limonene and pinene degradation (ko00903), as well as arginine and proline metabolism (ko00330) (Figure 3B—Contrasting tolerance).

On the other hand, in drought-tolerant scion stems (*T_S_*), the metabolic pathways of flavonoid biosynthesis (ko00941) and fructose and mannose metabolism (ko00520) were significantly enriched when grafted onto drought-sensitive rootstocks in the *S_S_*/S_R_ vs. *T_S_*/S_R_ comparison. In contrast, glucosinolate biosynthesis (ko00966) and nicotinate and nicotinamide metabolism (ko00760) pathways were enriched when grafted onto drought-tolerant rootstocks in the *S_S_*/T_R_ vs. *T_S_*/T_R_ comparison (Figure 3B—Contrasting tolerance).

#### 2.4.2. Comparisons among Rootstock Stems with Contrasting Drought Tolerance (S_R_ vs. T_R_): S_S_/S_R_ vs. S_S_/T_R_ and T_S_/S_R_ vs. T_S_/T_R_

The most enriched GO terms were identified in stems of drought-sensitive rootstocks (*S_R_*) grafted with drought-sensitive scions in the comparison S_S_/*S_R_* vs. S_S_/*T_R_*. These enriched terms were predominantly related to cell communication (GO:0007154), stress response (GO:0006950), and responses to various stimuli, including external (GO:0009605), abiotic (GO:0009628), biotic (GO:0009607), and chemical (GO:0042221) stimuli (Figure 3A—Contrasting tolerance).

MapMan analysis revealed an overrepresentation of stress-related BINs (BIN 20), including abiotic stress (BIN 20.2) in drought-sensitive rootstock stems (*S_R_*) grafted with sensitive scions, S_S_/*S_R_*. However, in drought-tolerant rootstock stems (*T_R_*), the only overrepresented group included TFs of the MADS-box family (BIN 27.3.24) (Table S6).

KEGG pathway analysis of comparisons between different rootstock stems (*S_R_* vs. *T_R_*) revealed mainly overrepresented pathways in drought-tolerant rootstock stems (*T_R_*). Riboflavin metabolism (ko00740) was enriched in drought-tolerant rootstock stems, and sesquiterpenoid and triterpenoid biosynthesis (ko00909) was enriched in drought-sensitive rootstock stems, regardless of the scion genotype (Figure 3B—Contrasting tolerance). Furthermore, when drought-tolerant rootstocks (*T_R_*) were grafted with drought-tolerant scions (T_S_/*T_R_*), overrepresented pathways included starch and sucrose metabolism (ko00500), pentose and glucuronate interconversions (ko00040), and steroid hormone biosynthesis (ko00140) (Figure 3B—Contrasting tolerance).

### 2.5. Analysis of Scion and Rootstock Genotype Interaction

The objective of this analysis was to explore modifications in the transcriptional profiles of scion (*S_S_* or *T_S_*) and rootstock (*S_R_* or *T_R_*) stems due to the effect of the grafted genotype.

#### 2.5.1. Transcriptomic Responses of Scions: S_S_/S_R_ vs. S_S_/T_R_ and T_S_/S_R_ vs. T_S_/T_R_

Analysis of changes in the transcriptional profiles of drought-sensitive (*S_S_* vs. *S_S_*) or drought-tolerant (*T_S_* vs. *T_S_*) scion stems by the effect of the grafted-rootstock genotype (S_R_ or T_R_) was performed by *S_S_*/S_R_ vs. *S_S_*/T_R_ and *T_S_*/S_R_ vs. *T_S_*/T_R_ comparisons.

The most significantly overrepresented GO and MapMan categories were identified in drought-sensitive scion stems (*S_S_*) when grafted onto drought-sensitive rootstocks (S_R_). The effect of drought-sensitive rootstocks (S_R_) could regulate the expression of genes in drought-sensitive scion stems (*S_S_*) associated with the stress response (GO:0006950; BIN 20), and response to external (GO:0009605) and biotic stimuli (GO:000960; PR-proteins, BINs 20.1 and 20.1.7). In addition, it could affect the expression of genes involved in cellular metabolic processes (GO:0044237) such as the metabolism of nitrogen compounds (GO:0006807) and cell wall degradation and modification (BINs 10.6 and 10.7) (Figure 3A—Genotype effect and Table S4). Furthermore, increased expression of transcription factors from the AP2/EREBP superfamily (BIN 27.3.3) was identified in drought-sensitive scion stems (*S_S_*) of S_S_/S_R_ grafts (Table S4).

In the analysis of KEGG metabolic pathways, no significant effect of the drought-sensitive rootstocks (S_R_) on drought-sensitive scion stems (*S_S_*) was identified. However, overrepresented KEGG pathways were identified in drought-sensitive scion stems (*S_S_*), potentially modulated by drought-tolerant rootstocks (T_R_). These groups were associated with pathways such as the monoterpenoid biosynthesis (ko00902) and metabolism of the arachidonic acid (ko00590), tryptophan (ko00380), or xenobiotics and drug metabolism by cytochrome P450 (ko00980) (Figure 3B—Genotype effect).

#### 2.5.2. Transcriptomic Responses of Rootstocks: S_S_/S_R_ vs. T_S_/S_R_ or S_S_/T_R_ vs. T_S_/T_R_

Analysis of modifications in transcriptional profiles of drought-sensitive (*S_R_* vs. *S_R_*) or drought-tolerant (*T_R_* vs. *T_R_*) rootstock stems by the effect of the grafted-scion genotype (S_S_ or T_S_) was performed by S_S_/*S_R_* vs. T_S_/*S_R_* and S_S_/*T_R_* vs. T_S_/*T_R_* comparisons. Only thirteen DEGs were identified in the S_S_/*T_R_* vs. T_S_/*T_R_* comparison: one down-regulated and twelve up-regulated (Figure 2E,F; Table S5). No overrepresented GO terms, MapMan BINs nor KEGG pathways were observed in this comparison, indicating little or no effect of scion genotypes on tolerant rootstock stems. Among the up-regulated DEGs, the genes encoding the gamma-1 regulatory subunit of the SNF1-related protein kinase (KING1: unigene183724) and cytochrome P450 750A1 (PUT-3641) stood out.

### 2.6. Gene Selection and Expression Analysis

#### 2.6.1. Gene Selection of Drought-Sensitive Genotypes

A total of 664 DEGs were associated with Gal 1056 scions, the drought-sensitive genotype (S_S_), or with both drought-sensitive genotypes (Gal 1056: S_S_ and R1S: S_R_) (Table S6). Among these DEGs, 33 were expressed exclusively in drought-sensitive scion stems (S_S_) and encoded proteins such as disease-resistance proteins (isotig28444, isotig47614, isotig58595, isotig61339, isotig73809, and unigene109681), glutamine synthases (unigene147662 and unigene147663), protein xyloglucan endotransglucosylase/hydrolase (PUT-1714), 12.5 kDa auxin-repressed protein (unigene208476), alcohol dehydrogenase 1 (isotig92113), lipase-like PAD4 (isotig73636), and ERFs (ERF016: unigene13714; ERF018: isotig62735) (Table S6). In both drought-sensitive genotypes (S_S_ and S_R_), 53 DEGs were exclusively expressed. These DEGs encoded proteins were associated with the regulation of transcription, including DNA topoisomerases 1 (isotig87308 and unigene22182), DNA-directed RNA polymerases (isotig78880 and isotig59976), and B-box zinc finger protein 19 (isotig08628). In addition, several genes encoded proteins involved in plant growth, such as TPX2 (unigene14564), EXORDIUM-LIKE 2 (isotig48717), and expansin B6 (unigene49640). Other DEGs encoded the gamma-humulene synthase (isotig84830), taxadiene synthases (unigene37488 and isotig50931), and dehydrin 2 (unigene4450) (Table S6).

The expression of 279 DEGs in the drought-sensitive scion stems (*S_S_*) could have been regulated by drought-sensitive rootstocks (*S*_R_). Further exploration of these DEGs in all comparisons revealed that the main effect of drought-sensitive rootstocks was the higher accumulation of transcript in drought-sensitive scion stems compared to drought-tolerant scion stems (*S_S_*/S_R_ vs. *T_S_*/S_R_), as well as in drought-sensitive rootstocks at the graft level (S_S_/S_R_—*S_S_* vs. *S_R_*). After filtering, a total of 43 DEGs remained, among which, 21 DEGs were exclusively expressed in drought-sensitive scion stems (*S_S_*) grafted on sensitive rootstocks (*S*_R_). Among the 43 DEGs, 13 genes encoded proteins associated with the biotic stress response such as disease resistance proteins RRS1 (WRKY 52: unigene12661 and unigene126345) (Table S7). In addition, other DEGs encoded proteins involved in plant growth such as P450-dependent fatty acid omega-hydroxylase (isotig47876), armadillo repeat only 1 protein (unigene189868, unigene98051, and unigene104325), cellulose synthase (unigene101434), xyloglucan glycosyltransferases (isotig27682 and unigene145925), cell wall beta-fructosidase 3 (isotig25901), and EXORDIUM (isotig126746). Other DEGs regulated by drought-sensitive rootstocks encoded transcription factors from the bHLH (bHLH95; unigene98089 and isotig57037), WRKY (WRKY62: isotig52323; WRKY164: isotig89865; and WRKY52: unigene12661, unigene145520, and unigene126345) and LOB (LOB6: isotig36282) families (Table S7).

The expression of 57 DEGs in drought-sensitive scion stems (*S_S_*) may also have been regulated by the effects of drought-tolerant rootstocks in *S_S_*/T_R_ grafts (Table S8). The main effect of the drought-tolerant rootstocks appeared to regulate the expression of 53 genes, resulting in equal expression levels in stems of both drought-sensitive and drought-tolerant scions (*S_S_*/T_R_ vs. *T_S_*/T_R_), and drought-tolerant rootstocks (S_S_/T_R_—*S_S_* vs. *T_R_*). Among these DEGs, some notable genes encoded proteins such as dirigent protein 11 (isotig18421 and isotig60288), probable aquaporin PIP2-8 (isotig50073), probable proline transporter 2 (isotig47139), aspartic proteinase (isotig29082), flavonol synthase/flavanone 3-hydroxylase (FLS1: unigene127279 and PUT-12904), and WAT1-related protein At1g68170 (isotig95747) (Table S8).

#### 2.6.2. Gene Selection of Drought-Tolerant Genotypes

A total of 609 DEGs were associated with either the drought-tolerant scion (Oria6: T_S_) or both drought-tolerant genotypes (Oria6: T_S_; and R18T: T_R_) (Table S9). Among them, 31 DEGs were exclusively expressed in T_S_ scions, while 18 DEGs were expressed in both drought-tolerant genotypes.

DEGs identified exclusively in the drought-tolerant scion (T_S_) encoded proteins such as the F-box protein PP2 (isotig49845 and unigene166508), bifunctional levopimaradiene synthases (unigene21053 and unigene107753), diterpene synthase (isotig53018), taxadiene synthases (isotig52219, unigene114035, and unigene206362), longifolene synthase (unigene112814), ferrochelatase-2 (unigene35391), auxin-responsive protein IAA13 (isotig112326), caffeoyl-CoA O-methyltransferase (unigene36622), salt tolerance receptor-like cytoplasmic kinases 1 (isotig88652 and unigene102915), and Hsp70-Hsp90 organizing protein 3 (unigene36612) (Table S9).

The DEGs identified in both drought-tolerant genotypes encoded proteins such as the small chain of the ribulose bisphosphate carboxylases (isotig106632 and unigene145862), subtilisin-like protease SBT1.8 (unigene21604), (1→3)-beta-glucan endohydrolase (unigene145711), longifolene synthase (unigene112814), taxadiene synthase (isotig34500), BRI1-associated receptor kinase 1 homolog (isotig25758), phosphoglycerate kinase (unigene127991), probable aldo-keto reductase 1 (isotig83404) and 3 (isotig47827), probable protein phosphatase 2C 3 (PP2C03: unigene146886 and isotig47726), and serine/threonine-protein phosphatase 2A activator 2 (unigene511) (Table S9).

*T_S_*/S_R_ vs. *T_S_*/T_R_ comparison revealed no DEGs in drought-tolerant scion stems (*T_S_*) significantly associated with the effect of drought-sensitive rootstocks (S_R_) (Figure 2F). A specific DEG encoding NAC domain-containing protein 68 (isotig49137) in drought-tolerant scion stems that showed an association with drought-tolerant rootstocks (T_R_) was identified.

### 2.7. Analysis of Differentially Expressed Transcription Factors (DETFs)

**Figure 4:**
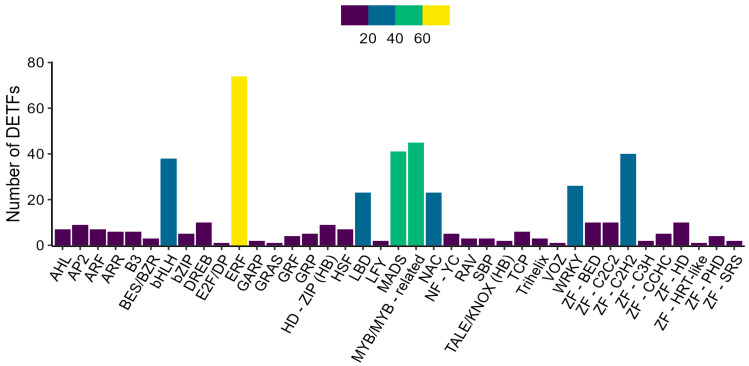
Classification of differentially expressed transcription factors (DEFTs) identified in all comparisons.

A total of 461 DETFs, classified into 39 families, were identified among all comparisons. DETFs were filtered into the top nine families (320 DETFs): bHLH, DREB, ERF, LBD, MADS, MYB/MYB-related, NAC, WRKY, and ZF-C2H2 (Figure 4). 

**Figure 5:**
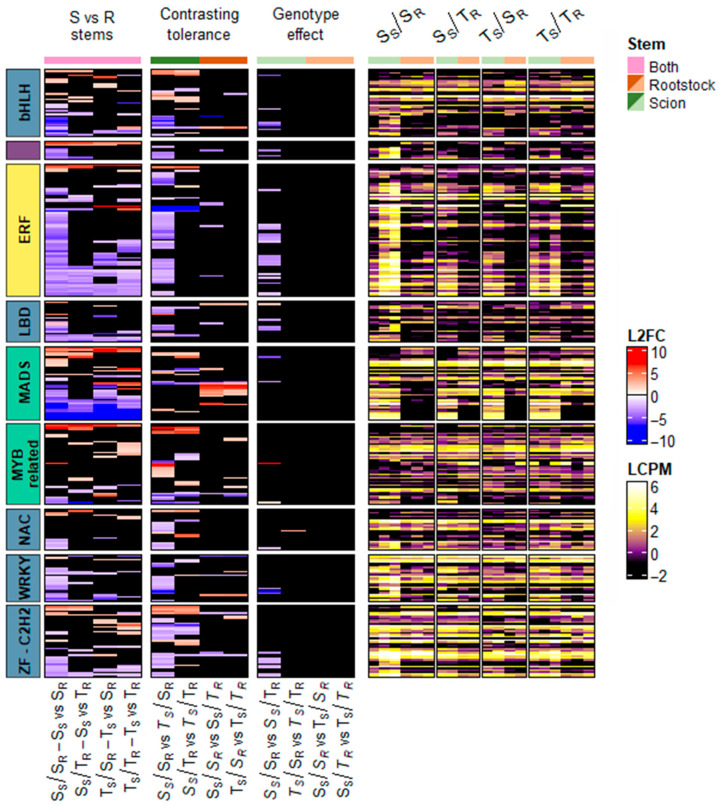
Distribution of the most abundant differentially expressed transcription factors (DEFTs) families and their normalized expression profiles by graft type. Log_2_ fold change (L2FC) in red indicates DEGs with higher expression in rootstock stems, drought-tolerant genotypes, or in stems grafted with the drought-tolerant genotype in S vs. R stems, Contrasting tolerance, or Genotype effect columns, respectively. In contrast, L2FC in blue indicates DEGs with higher expression in scion stems, drought-sensitive genotypes, or in stems grafted with the drought-sensitive genotype in S vs. R stems, Contrasting tolerance, or Genotype effect columns, respectively.

The ERF and MADS families exhibited the highest number of DETFs. In all graft combinations, scion stems showed a higher accumulation of DETFs from the ERF family, which is particularly evident in drought-sensitive scion stems of S_S_/S_R_ grafts. This pattern was also observed when analyzing the effect of drought-sensitive rootstocks on sensitive scion stems (Figure 5). The MADS family showed some DETFs with higher accumulation in scion stems and others in rootstock stems, and differences between genotypes were identified when comparing rootstock stems of contrasting genotypes (Figure 5).

Several DEGs stood out in all comparisons and were potentially associated with scion genotypes or phenotypes. Four DEGs were identified with increased transcript accumulation in scion stems, especially in the S_S_/S_R_ grafts: unigene12131 (MADS), unigene2726 (ERF), unigene17220 (ERF), and isotig43717 (ZF-C2H2). Other DEGs that showed increased transcript accumulation in the S_S_/S_R_ graft combination encoded ERF (isotig15113), ZF-C2H2 (isotig43717), and WRKY (unigene6388). In addition, certain DEGs, such as unigene13714 and isotig49919 (AP2-EREBP) or unigene6381 (ZF-C2H2), showed higher expression levels in scion stems (S_S_) or in both drought-sensitive genotypes, respectively. In contrast, we identified DETFs that were negatively correlated with drought-sensitive scions, including isotig12432 (ZF-C2H2), and with drought-tolerant scions, such as isotig10262 (ZF-CCHC) and unigene16552 (ZF-C2H2).

### 2.8. Gene Expression Analysis by Quantitative Real-Time PCR

**Figure 6:**
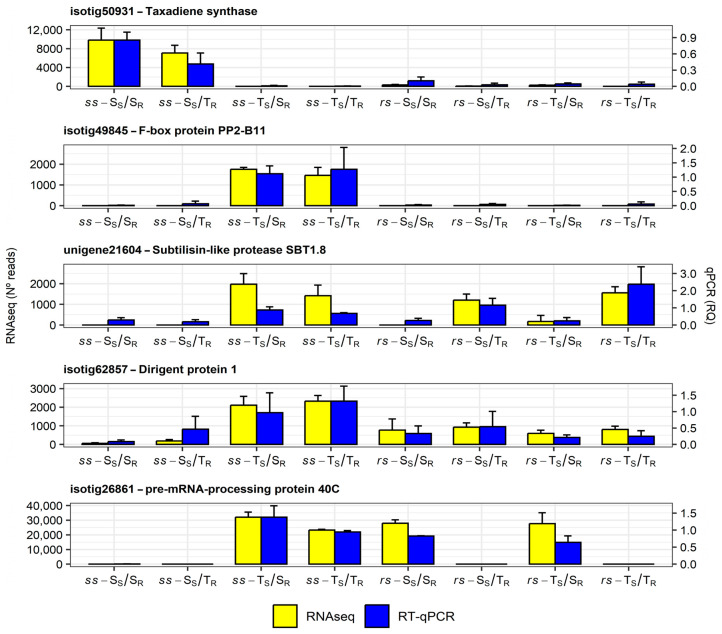
Validation of sequencing data of scion (*ss*) and rootstock (*rs*) stems of *P. pinaster* grafts by RT-qPCR. Relative quantification (RQ) by RT-qPCR (blue) and mean RNA-Seq expression values (yellow) of five selected DEGs associated with scion and rootstock genotypes, and effect of graft combination.

The relative quantification of selected DEGs showed results concordant with previous transcriptomic analyses. The expression patterns of DEGs encoding taxadiene synthase (isotig50931) and F-box protein PP2-B11 (isotig49845) were associated with drought-sensitive (S_S_) and drought-tolerant scion genotypes (T_S_), respectively (Figure 6). The subtilisin-like protease SBT1.8 (unigene21604) had an increased expression in both drought-tolerant genotypes (T_S_ and T_R_). Both Dirigent protein 1 (isotig62857) and pre-mRNA-processing protein 40C (isotig 26861) had an increased expression in drought-tolerant scion stems (T_S_), but the former had a similar expression between rootstock genotypes (S_R_ and T_R_) and the latter only in the drought-sensitive genotype (S_R_) (Figure 6).

## 3. Discussion

### 3.1. Expression of Genes Involved in Pathogen Recognition in Pinus Pinaster Grafts Depended on Scion Provenance and Genotype Combination

**Figure 7:**
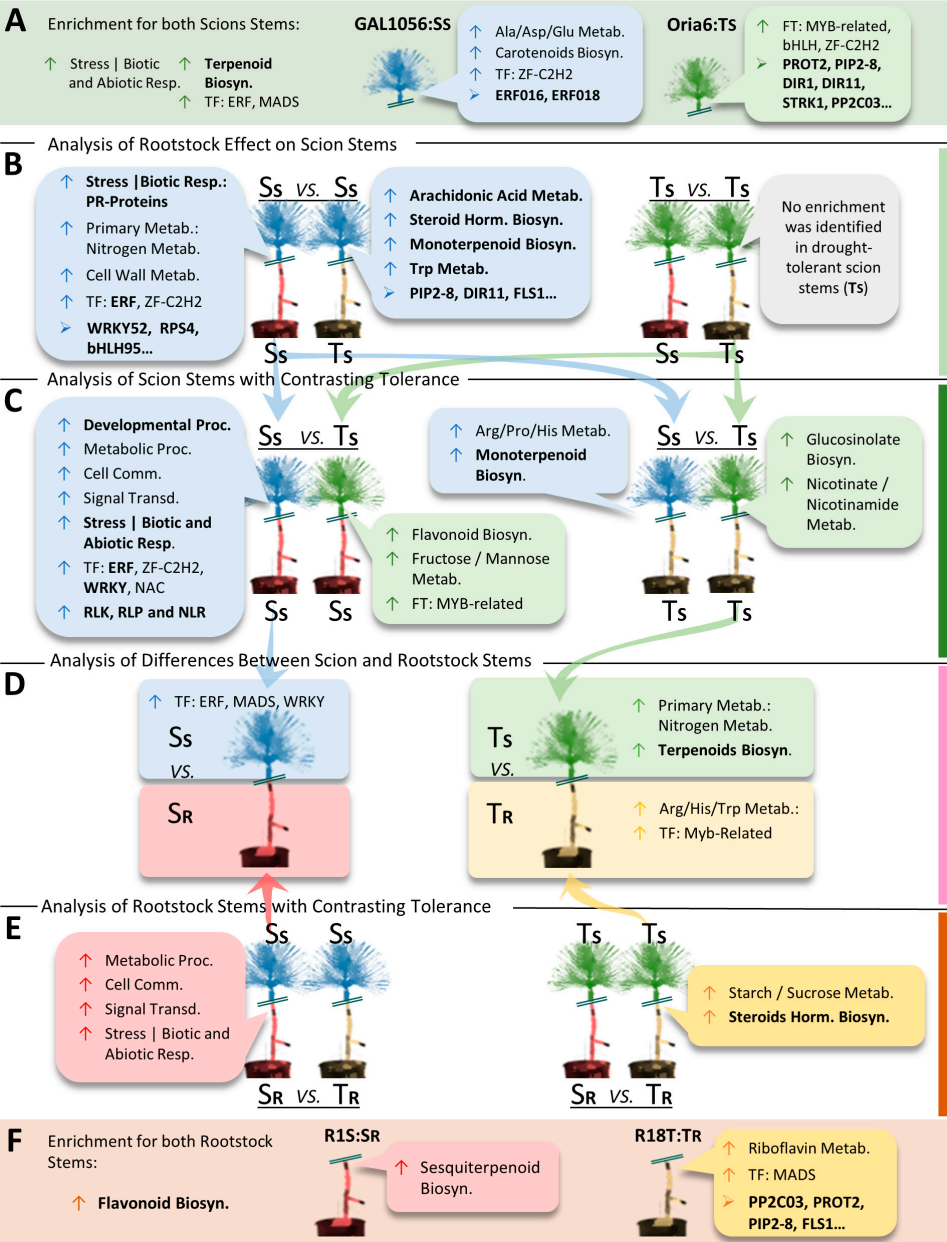
Schematic representation of graft-induced transcriptome dynamics. Graphical summary of (**A**,**D**,**F**) transcript profile variation between scion and rootstock stems of each graft of *P. pinaster*; (**B**) Effect of rootstock on scion stems: comparison of drought-sensitive (S_S_) or drought-tolerant (T_S_) scion stems grafted on rootstocks with contrasting drought response; transcript profile of scions and rootstock stems with contrasting drought tolerance: comparison of drought-sensitive (S_S_) vs. drought-tolerant (T_S_) scion stems (**C**), or drought-sensitive (S_R_) vs. drought-tolerant (T_R_) rootstock stems (**E**). Vertical (↑) and horizontal (>) arrows indicate enriched clusters and highlighted genes in the analyzed stem.

Plants, as long-lived sessile organisms, have developed intricate systems to recognize and respond to numerous external stimuli, including environmental disturbances. *Pinus pinaster* populations in the Iberian Peninsula show different local adaptations to biotic and abiotic stresses, such as drought or pathogen attack [10,11,12,14]. Resource availability plays a significant role in shaping genetic patterns of adaptive variation among populations, giving rise to locally adapted defensive strategies indirectly mediated by resource availability, such as those against herbivore pressure [45]. Pinaceae species, such as *P. pinaster*, have evolved diverse strategies to cope with pathogen attacks that depend on their growth rate and the availability of resources. Fast-growing *P. pinaster* trees invest more resources in inducible defenses, while slow-growing trees invest more in constitutive defenses [46,47]. In the stems of drought-sensitive scions of *P. pinaster* grafts, numerous DEGs encoding proteins associated with pathogen recognition and the activation of plant immunity were identified. Those DEGs included genes encoding receptor-like kinases (RLK) and receptor-like proteins (RLP), as well as nucleotide-binding domain and leucine-rich repeat receptors (NRL o NB-LRR) (Figure 7B,C), which are involved in pattern- and effector-triggered immunity, respectively [48,49]. NRL receptors were abundant in drought-sensitive scion stems and included disease-resistant proteins such as RUN1, TAO1, or RPS4. NLRs are cytoplasmic receptors that detect interference from pathogen virulence factors (effectors) leading to the activation of the effector-triggered immunity [50]. Pines exhibit high diversity in NLR gene sequences [51] and several NLRs play a role in plant resistance to pathogens such as the *Cronartium genus* in *P. monticola*, *P. flexilis,* and *P. taeda* [52,53,54], or *B. xylophilus* and *F. circinatum* in *P. pinaster* [55,56] and *P. radiata* [57].

Drought-sensitive and drought-tolerant scion donor trees exhibit contrasting growth rates, with Gal 1056, a drought-sensitive scion donor, being a plus tree for wood production. Differential accumulation of DEGs involved in responses to biotic stress and external stimuli between scion stems may be associated with growth-defense genetic trade-offs of scion genotypes (Figure 7C). A similar growth trade-off was suggested in *Abies pinsapo* and *Cedrus atlantica* seedlings under drought conditions [58,59]. Furthermore, the number of DEGs associated with biotic stress and responses to external stimuli was higher when scions from drought-sensitive rootstocks were grafted onto drought-sensitive rootstocks in S_S_/S_R_ grafts (Figure 7B). These results indicate that the regulation of gene expression in *P. pinaster* scion stems, in relation to biotic stress perception and signaling, depends on rootstock genotype and grafting combination. López-Hinojosa et al. described a similar transcriptional pattern in needles of the same *P. pinaster* S_S_/S_R_ grafts [44].

Water availability and climate conditions also influence defense-related gene patterns. The oceanic Atlantic climate of north-western Spain favors the spread and constant attacks of plant pathogens such as *F. circinatum* [60,61]. Consequently, the drought-sensitive Gal 1056 (*S_S_*) progenitor may have acquired responses to cope with such attacks, based on constitutive expression of genes associated with plant-inducible defenses, as a local adaptation to its provenance, as well as to nutrient availability and growth.

### 3.2. Drought Susceptibility of P. Pinaster Grafts May Be Influenced by the Expression of Genes Involved in the Response to Biotic Stress

Biotic and abiotic stress responses are closely related, and several genes associated with biotic stress responses have been identified in abiotic stress responses. For example, DEGs encoding NRL proteins have been identified under drought treatments, suggesting that transcriptional regulation of NLR-encoding genes is not only related to pathogen response and resistance but also to drought response and tolerance [51,62]. In particular, LRR receptor-like protein kinases have been associated with molecular mechanisms underlying abiotic stress responses, including drought [63]. Thus, the identified proteins related to biotic stress signaling could also be involved in the drought responses and tolerance of *P. pinaster*.

In our case, we identified several proteins in drought-sensitive scion stems (*S_S_*) which may be associated with its response and sensitivity to drought (Figure 7B,C). For example, the disease resistance proteins RRS1 (unigene126345 and unigene12661), also known as probable transcription factor WRKY52, is a factor involved in the up-regulation of disease resistance genes prior to growth arrest and necrosis development under low humidity conditions in Arabidopsis [64]. Another protein of interest is the nuclear immune receptor RPS4 (unigene31318 and unigene2411), which is required for RRS1-dependent constitutive defense activation in Arabidopsis [65], and was also found to have increased transcript accumulation in S_S_, possibly due to the effect of drought-sensitive rootstocks (Figure 7B).

In addition, certain differentially expressed transcription factors could be associated with the effects of drought-sensitive rootstocks on sensitive scions. For example, WRKY62 (isotig52323), is an SA-regulated WRKY that controls the transcription of defense-related genes [66]. In drought-sensitive scion stems or drought-sensitive genotypes, two differentially expressed transcription factors, unigene13714 (ERF016) and isotig62735 (ERF018), could be associated with the response of drought-sensitive scion stems (Figure 7A). Mapman annotation revealed that these DEGs are related to AT5G21960, a member of the A-5 subfamily of DREB (ERF subfamily Group II). A phylogenetic analysis of Arabidopsis AP2/EREF TFs showed that those DEGs and the DREB RAP2.1 (AT1G46768) were phylogenetically related [67]. RAP2.1 (AT1G46768) is a member of subgroup A-5 of the DREB subfamily that accumulates in response to drought stress. Dong and Lui suggested that RAP2.1 might act as a negative regulator involved in the precise regulation of stress-related genes, and the high level of expression negatively regulates plant tolerance to drought stress [68]. Therefore, these DEGs could be involved in the drought response of drought-sensitive scion stems.

### 3.3. The Expression of Drought Tolerance Genes in P. pinaster Stems Could Predate the Drought Event in Tolerant Genotypes

The phytohormone abscisic acid (ABA) is a signaling molecule that plays a crucial role in both biotic and abiotic stress responses. Under drought conditions, roots sense soil water deficits and ABA is transported through the xylem from roots to target cells where it induces cellular responses to drought, leading to modifications in cellular, metabolic, and transcriptomic profiles [69,70].

In this study, several ABA-related transcripts were identified such as bHLH95 (isotig57037 and unigene98089) (Figure 7B), a transcription factor involved in the regulation of abscisic acid biosynthesis [71], or PP2C (probable protein phosphatase 2C) proteins, such as PP2C03 (unigene146886 and isotig47726) (Figure 7A,F). PP2C proteins are one of the major families of protein phosphatases in plants that play important roles in different pathways regulating plant growth regulatory signaling pathways such as ABA, BR, and the MAPK cascade in plant response to abiotic stress [72,73]. ABA-mediated drought signaling is regulated by the ABAR-PP2C-SnRK2 module. Therefore, the PP2C03 proteins could be part of the signaling that modulates the drought response of sensitive and tolerant scions (Figure 7A,F).

Other DEGs were found to potentially modulate drought tolerance, such as the probable proline transporter 2 (PROT2: isotig47139) and the probable aquaporin PIP2-8 (isotig50073), with both proteins having a higher accumulation in drought-tolerant rootstocks (Figure 7A,F). These proteins are associated with compatible solute transport, nitrogen, and water distribution that could help maintain cell turgor during water restriction, potentially increasing tolerance to hydric stress during drought events [74,75,76].

In addition, genes encoding the protein salt tolerance receptor-like cytoplasmic kinases 1 (STRK1: isotig88652 and unigene102915) were identified (Figure 7A). This RLCK receptor is up-regulated during stress conditions such as cold, dehydration, and salt, and is associated with the activation of proteins such as catalases, which have ROS-scavenging activity [77,78]. The presence of this type of receptor could be associated with the increased drought tolerance of drought-tolerant scions or their perception of drought stress.

### 3.4. Drought-Tolerant Rootstock (R18T) Controlled the Expression of Metabolism-Related Genes in Gal 1056 Stems

Previous studies on the same progeny have revealed that high levels of metabolites and transcripts related to drought response are present in drought-tolerant genotypes even before water deficit occurs [43,79,80]. KEGG analysis in this study revealed groups overrepresented in drought-sensitive scion stems when grafted onto drought-tolerant rootstocks of S_S_/T_R_ grafts. Those groups were associated with lipid metabolism (arachidonic acid and steroid hormones), secondary metabolites (monoterpenoids, caffeine, and retinol), tryptophan, and xenobiotics (Figure 7B).

The metabolic pathway of arachidonic acid, a fatty acid component of the cell membrane, was enriched in drought-tolerant *P. pinaster* genotypes, along with inositol phosphate [80]. Increased accumulation or external application of arachidonic acid precursors has been associated with increased drought resistance through responses to ABA, as well as a decreased leaf area and biomass accumulation [81]. In addition, arachidonic acid also contributes to maintaining membrane integrity and the functionality of integral membrane proteins under stress [82].

Brassinosteroids (BRs) are steroid hormones that play important roles in plant growth, development, and response to stresses such as drought [83,84]. In conifers, such as *Picea abies* and *Pinus massoniana*, BRs were associated with the regulation of drought tolerance and growth, and lignin synthesis and xylem development, respectively [85,86]. DEGs encoding 11-oxo-beta-amyrin 30-oxidases (unigene138352 and unigene196133) and cytochrome P450 734A1 (unigene112417) were found in both arachidonic acid and brassinosteroid metabolism. In addition, 11-oxo-beta-amyrin 30-oxidase and cytochrome P450 734A1 were also involved in the biosynthesis of triterpenoid saponins [87], and in the regulation of BR-inactivation/homeostasis and plant growth [88], respectively.

In conifers, terpenoid biosynthesis has been found to play an important role in the responses to both abiotic and biotic stresses, as well as in local adaptation to environmental conditions [43,58,59,89]. Terpenoids are stored in the resiniferous ducts, serving as a defense mechanism to protect the plant from invading pathogens and herbivores. In addition, some terpenes exhibit antioxidant properties, indicating their potential role in counteracting oxidative stress induced by internal and external stimuli [90]. The accumulation of gene transcripts associated with monoterpenoid biosynthesis was found to increase in drought-sensitive scion stems when grafted with drought-tolerant rootstocks (Figure 7B,C). In *Pinus sylvestris*, monoterpenes accumulate under drought events [91]. Terpenoid profiling showed little difference in monoterpenoid accumulation in scion stems, but their concentration increased slightly in rootstock stems under water deficit conditions [43]. Therefore, their accumulation could be associated with a drought response of rootstock stems. Fernández de Simón et al. revealed that diterpenes are the most abundant terpenoid in grafted stems of *P. pinaster* under water deficit conditions [43]. In addition, they detected that scion and rootstock stems accumulate low amounts of terpenes compared to needles and roots, indicating an organ- and genotype-dependent regulation of terpenoid biosynthesis. In this study, we confirmed, at the transcriptional level, that scion stems exhibited a higher accumulation of DEGs associated with terpenoid biosynthesis (Figure 7A), with higher expression in drought-sensitive scions grafted onto drought-sensitive rootstocks during well-watered conditions, which is consistent with the terpene profiles of *P. pinaster* grafts [43]. Therefore, the identified DEGs associated with terpenoid biosynthesis such as taxadiene synthase, longifolene synthase chloroplastic, or geranylgeranyl pyrophosphate synthase could be important in terpene accumulation during well-watered and drought conditions in tolerant genotypes. Dirigent proteins showed an increased transcript accumulation in drought-tolerant scion stems (DIR1) (Figure 7A), or both drought-tolerant and drought-sensitive scion stems grafted onto drought-tolerant rootstocks (DIR11) (Figure 7A,B). These proteins are involved in lignan and lignin biosynthesis. In addition, DIR1 expression increases in response to osmotic stress, whereas DIR11 responds to biotic stress such as *Pseudomonas syringae* infection [92]. Other DEGs such as the caffeoyl-CoA O-methyltransferase (unigene36622) involved in lignin biosynthesis were identified in drought-tolerant scion stems [93]. Therefore, lignan and lignin biosynthesis could also be important pathways in the response of maritime pine to drought.

Several DEGs were involved in the biosynthesis of flavonoids, which are polyphenols that play an essential role in reducing ROS-oxidative-damage stress events [94,95]. DEGs associated with flavonoid metabolism were overrepresented in rootstock stems (Figure 7F) as well as in tolerant scions grafted onto sensitive rootstocks. Therefore, the regulation of their expression in scion stems could be modulated by the rootstock genotype; this may be the case for flavonol synthase/flavanone 3-hydroxylase (FLS1: PUT-12904 and unigene127279) (Figure 7F), which contributed to the flavonol biosynthesis [96], regulated by the accumulation of the auxin indole-3-acetic acid (IAA) [97]. The presence of auxin transporters, such as auxin transporter-like protein 4 (isotig59937), further suggests a possible synergy between those DEGs.

## 4. Material and Methods

### 4.1. Plant Material and Experimental Design

Four grafting constructions were designed and grafted at the Centro de Mejora Genética Forestal de Valsaín (Segovia, Spain). The grafts combined scions and rootstocks of *Pinus pinaster* genotypes with contrasting drought tolerance (for more information see de Miguel et al. 2012, 2014 [98,99]). Two genotypes from different populations, Gal 1056 and Oria 6, were used as scions. Gal 1056, a highly drought-sensitive (S_S_) elite pine, belongs to a breeding program from north-western Spain (Pontevedra, 42°10′ N 8°30′ W) where the weather is usually mild and humid throughout the year. Oria 6 is a drought-tolerant (T_S_) individual from Sierra de Oria, a natural population in south-eastern Spain (Almería, 37°31′ N 2°21′ W), which suffers from recurrent drought events and temperature variation. To maximize compatibility between scion and rootstock combinations, two F1 siblings from a controlled cross Gal 1056 × Oria 6 were used as rootstocks. The rootstocks were R1S (drought-sensitive rootstock, S_R_) and R18T (drought-tolerant rootstock, T_R_). The F1 genotypes were vegetatively propagated, and three-year old plants were used as rootstocks. The four grafts designed were Gal 1056/R1S (S_S_/S_R_), Gal 1056/R18T (S_S_/T_R_), Oria 6/R1S (T_S_/S_R_), and Oria 6/R18T (T_S_/T_R_), each represented by three replicates (Scheme 1A).

Eight months after top-grafting, the trees were grown in a climate walk-in chamber (Fitoclima 10000EHHF, Aralab, Rio de Mouro, Portugal) for six months under controlled conditions as described by López-Hinojosa et al. [44]. Briefly, the average temperature and relative humidity settings were 25 °C and 65% during the light photoperiod (14 h), and 20 °C and 60% during the dark photoperiod (10 h). Grafted trees were watered to field capacity, maintaining the soil volumetric water content at 20%. A randomized block design was applied to prevent systematic errors such as the edge effect, and grafts were periodically redistributed randomly among blocks once per week [100]. The phenotypic evaluation of the grafted plants has been conducted and described in Fernández de Simón et al. [43]. Scion and rootstock stems were sampled from 2.5 cm above and below each graft junction. After collection, samples were frozen in liquid nitrogen and stored at −80 °C until RNA extraction.

### 4.2. RNA Extraction, RNA-Seq Library Preparation, and Sequencing

Frozen stems were homogenized using an IKA^®^ A11 basic analytical mill (IKA-Werke GmbH & Co. KG, Wilmington, NC, USA). Total RNA was extracted from each powdered scion and rootstock stem sample using the Plant/Fungi Total RNA Purification^®^ Kit (Norgen Biotek Corp., Thorold, ON, Canada) following the manufacturer’s instructions. RNA integrity, concentration, and quality were assessed using 1% (*w*/*v*) agarose gel and a NanoDrop One spectrophotometer (NanoDrop Technologies, Wilmington, DE, USA). Twenty-four cDNA libraries were prepared using the Illumina TruSeq Stranded mRNA LT Sample Preparation Kit and paired-end sequenced using Illumina NovaSeq 6000 by Macrogen (Seoul, Republic of Korea).

### 4.3. Sequence Analysis and Transcript Abundance Estimation

Quality checks of the raw sequence data were performed using the quality check tool implemented in OmicsBox software (version 2.0.36) [101], based on FastQC software (version 0.11.9) [102]. Universal adapters were removed from the raw paired-end sequences using the FASTQ pre-processing tool Trimmomatic (version 0.38) [103] implemented in the OmicsBox software. The Reformat.sh script from the BBTools (version 38.90) pack was used to trim the read-end nucleotides and regions with a mean quality score of <20, and to filter out the sequences with a minimum length of <30 bp and a minimum mean quality of <20 (https://sourceforge.net/projects/bbmap/; accessed on 9 October 2020). Next, the SortMeRNA tool (version 4.2.0) was used to remove rRNA from reads, specifying the - - *paired_in* option to remove both paired-end reads if one of them matched a sequence from the rRNA databases [104]. The Salmon program (version 1.4.0) was used to map clean paired-end sequences to the *P. pinaster* reference transcriptome and to quantify the relative number of sequences per transcript [105].

### 4.4. Differential Expression Analysis

Differential expression analysis was performed using DESeq2 (version 1.34.0) in R, according to the procedures described by Love et al. [106]. Genes considered as differentially expressed showed an adjusted *p* value < 0.05 and log_2_fold change > 1.5 or < −1.5. Twelve differential expression analyses were carried out to unravel the transcriptional and functional profiles that might be affected by factors inherent to the chimeric pines (Scheme 1B). For comparisons, we refer to the stem (scion and/or rootstock) analyzed in italics. The specific objectives of each analysis were as follows:

1. Analysis of the transcriptional differences between scion and rootstock stems of each *P. pinaster* graft combining genotypes with similar (S_S_/S_R_ and T_S_/T_R_) or contrasting (S_S_/T_R_ and T_S_/S_R_) responses to drought. The comparisons were S_S_/S_R_: Gal 1056 vs. R1S, T_S_/T_R_: Oria 6 vs. R18T, S_S_/T_R_: Gal 1056 vs. R18T, and T_S_/S_R_: Oria 6 vs. R1S (Scheme 1B).
2. Analysis of the transcriptional variation between scion stems. Two types of comparisons were included: (1) identification of differentially expressed genes (DEGs) between drought-sensitive and drought-tolerant scion stems (S_S_ vs. T_S_) grafted on the same rootstock genotype (*S_S_*/S_R_ vs. *T_S_*/S_R_: Gal 1056/R1S vs. Oria 6/R1S and *S_S_*/T_R_ vs. *T_S_*/T_R_: Gal 1056/R18T vs. Oria 6/R18T); and (2) identification of DEGs in drought-sensitive (S_S_) or drought-tolerant (T_S_) scion stems grafted on different rootstock genotypes, and therefore associated with rootstock interactions (*S_S_*/S_R_ vs. *S_S_*/T_R_: Gal 1056/R1S vs. Gal 1056/R18T and *T_S_*/S_R_ vs. *T_S_*/T_R_: Oria 6/R1S vs. Oria 6/R18T) (Scheme 1B).
3. Analysis of the transcriptional variation between rootstock stems. Two types of comparisons were included: 1) identification of DEGs between rootstock genotypes (S_R_ vs. T_R_) with the same grafted scion: S_S_/*S_R_* vs. S_S_/*T_R_*: Gal 1056/R1S vs. Gal 1056/R18T and T_S_/*S_R_* vs. T_S_/*T_R_*: Oria 6/R1S vs. Oria 6/R18T); and 2) identification of DEGs in drought-sensitive (S_R_) or tolerant (T_R_) rootstock stems with different grafted scions, associated with scion interactions (S_S_/*S_R_* vs. T_S_/*S_R_*: Gal 1056/R1S vs. Oria 6/R1S and S_S_/*T_R_* vs. T_S_/*T_R_*: Gal 1056/R18T vs. Oria 6/R18T) (Scheme 1B).

### 4.5. Pinus pinaster Transcriptome Annotation and Functional Enrichment Analysis

The *P. pinaster* reference transcriptome (http://www.procogen.eu accessed on 16 May 2024) contains 206,575 transcripts. Functional annotation of transcripts was performed using multiple tools implemented in OmicsBox software. The BLASTx tool was used to annotate the *P. pinaster* reference transcriptome by aligning it against homologous sequences available in public databases such as NCBI non-redundant (nr), Swiss-Prot, or InterPro. Gene Ontology (GO) terms were assigned to the blasted results using the implemented tool Blast2GO (version 2021.0) [107]. The *E*-value threshold established was 10^−6^. KEGG Orthology and pathway assignments were performed using the tool implemented for the analysis of metabolic pathways [108].

Enrichment analyses of Gene Ontology terms and KEGG pathways were performed using the implemented tool for Fisher’s exact test for each comparison. The statistical parameters were FDR < 0.05 and a one-tailed analysis for GO results, and *p*-value < 0.05 and a two-tailed analysis for KEGG pathway results. The Mercator (version 3.6) annotation tool [109], available on the plaBi database website (https://plabipd.de/portal/mercator-sequence-annotation; accessed on 10 December 2021), was used to assign MapMan annotations (BINs), and MapMan desktop software was used to analyze the MapMan functional classification of the DEGs identified in the comparisons. The statistical analysis performed with MapMan desktop software is based on the Wilcoxon rank sum test, which established differential BIN distributions between up- and down-regulated DEGs.

### 4.6. Gene Selection by Correlation Analysis and Validation by qRT-PCR

To validate the transcriptomic study, the expression of five selected DEGs was analyzed by real-time RT-qPCR. The selection of genes associated with the stem genotype and graft combination was performed using the R package (version 4.1.3) WGCNA (weighted correlation network analysis) [110]. DEG-specific primers were designed using the NCBI Primer-BLAST tool (https://www.ncbi.nlm.nih.gov/tools/primer-blast/; accessed on 5 March 2024). Selected DEGs and their primers are listed in Table S10. RT-qPCR experiments were performed using three biological samples per genotype and three technical replicates each. The 18S rRNA transcript was used as an internal control to normalize the expression levels from different samples for quantitative transcript accumulation analysis. Synthesis of cDNA was performed from 1 μg of total RNA using SuperScript III First-Strand Synthesis System (Invitrogen by Thermo Fisher Scientific, Waltham, MA, USA) according to the manufacturer’s instructions. Polymerase chain reactions were performed in an Applied Biosystems 7500 Fast Real-Time PCR System (Applied Biosystems by Thermo Fisher Scientific, Waltham, MA, USA), using FastStart Universal SYBR Green Master (Rox; F. Hoffmann-La Roche Ltd., Basel, Switzerland).

The reactions contained 25 ng cDNA, 500 nM forward primer, 500 nM reverse primer, and 1× SYBR Green Master. They were subjected to an initial step of 10 min at 95 °C, followed by 40 cycles of 15 s at 95 °C and 60 s at 60 °C. A melting-curve analysis was included to verify the specificity of each primer. Relative quantification (RQ) was calculated automatically by the ΔΔCt method (RQ = 2^−ΔΔCt^; Ct = threshold cycle), where the first ΔCt is the difference between the Ct value of the internal control (Ri18S) and the Ct value of the selected DEG for each sample and ∆∆Ct represents the difference between the ∆Ct of each sample and the ∆Ct of a reference sample, using 7500 Software (version 2.3; Life Technologies by Thermo Fisher Scientific, Waltham, MA, USA).

## 5. Conclusions

The analysis of *P. pinaster* graft stems combining genotypes with contrasting responses to drought led us to characterize different expression patterns associated with responses to biotic and abiotic stress. The expression of genes involved in pathogen recognition in *P. pinaster* grafts was associated with scion provenance and graft combination. Genotypes used as scions showed differential accumulation of defense-related genes, suggesting genetic trade-offs between growth and defense. These trade-offs could not only affect defense accumulation at the expense of growth but also tolerance to water deficits. The combination of grafts proved to be crucial in obtaining the desired responses in stems. Drought-tolerant rootstocks showed an increased expression of metabolism-related genes, including arachidonic acid and brassinosteroid pathways, possibly contributing to increased drought tolerance. Furthermore, the accumulation of terpenoid-associated genes in *P. pinaster* grafts was mainly scion-dependent, whereas flavonoid accumulation was rootstock-dependent. This analysis reveals a novel insight into how genotype combination might affect the transcriptomic profile of pine stems, showing that DEGs are potentially associated with the response to the drought of stems, a not well-studied organ compared to needles and rootstock. Furthermore, it underscores grafting as a suitable approach for exploring the transcriptomic profiles across genotypes and their response to genotype combinations. This suggests grafting as a potential method for selecting genotypes for both scions and rootstocks, based on the desirable responses of the grafted conifers. Finally, considering that recurrent droughts have been affecting the western Mediterranean region during the last few years, the selection of drought-tolerant genotypes as rootstocks of *P. pinaster* grafting has been proven to be a useful strategy to improve the stress tolerance of drought-sensitive scions selected on the basis of growth rate, wood yield, and resistance to pests or pathogens.

## Data Availability

The data presented in this study are available on request from the corresponding author because the data are part of an ongoing study.

## Supplementary Materials

The following supporting information can be downloaded at: https://www.mdpi.com/article/10.3390/plants13121644/s1. Figure S1: Percentage of annotated sequences. Table S1: RNA-seq results of stem samples of *Pinus pinaster* grafts, Table S2: Mapping results from Salmon software. Table S3: Enrichment analysis of Mapman BINs from comparisons between scion and rootstock stems (S vs. R) of each *P. pinaster* graft. Table S4: Enrichment analysis of Mapman BINs from comparisons between scion stems of *P. pinaster* graft. Table S5: Enrichment analysis of Mapman BINs from comparisons between rootstock stems of *P. pinaster* graft. Table S6: Normalized-read counts of DEGs associated with genotypes of drought-sensitive scions (Gal 1056 scions: S_S_), or with both drought-sensitive genotypes (Gal 1056: S_S_; and R1S: S_R_). Table S7: Normalized-read counts of DEGs identified in drought-sensitive scion stems (S_S_) that could have been regulated by drought-sensitive rootstocks (S_R_) in S_S_/S_R_ grafts. Table S8: Normalized-read counts of DEGs identified in drought-sensitive scion stems (S_S_) that could have been regulated by drought-tolerant rootstocks (T_R_) in S_S_/T_R_ grafts. Table S9: Normalized-read counts of DEGs associated with associated with either drought-tolerant scions (Oria6: T_S_) or both drought-tolerant genotypes (Oria6: T_S_; and R18T: T_R_). Table S10: Primer sequences for RT-qPCR.
