# Supplementary figures and images for: Maritime Pine Rootstock Genotype Modulates Gene Expression Associated with Stress Tolerance in Grafted Stems
Source: Plants (Basel). 2024 Jun 14;13(12):1644. doi: 10.3390/plants13121644 (PMC11207801; doi:10.3390/plants13121644)

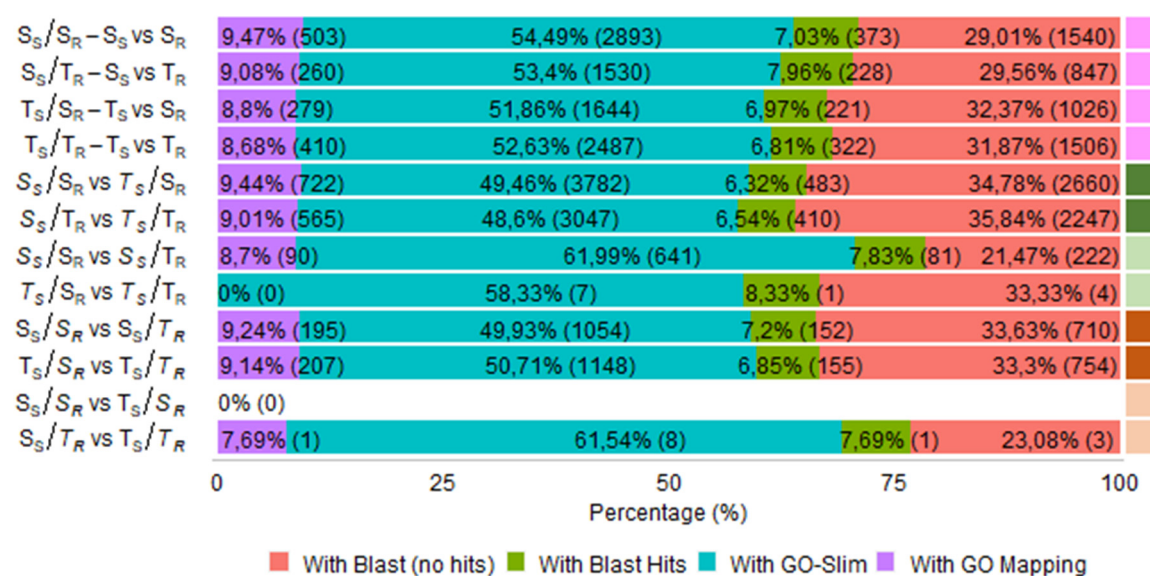

Figure S1. Percentage of annotated sequences.

Supplement: Supplementary file 1 [file plants-13-01644-s001.zip › plants-3039106-Figure S1.pdf]
